# Risk Estimation for Infection in Patients With ST-Segment Elevation Myocardial Infarction Undergoing Percutaneous Coronary Intervention: Development and Validation of a Predictive Score

**DOI:** 10.3389/fcvm.2022.845307

**Published:** 2022-04-15

**Authors:** Yuanhui Liu, Litao Wang, Pengyuan Chen, Yining Dai, Yaowang Lin, Wei Chen, Zhengrong Xu, Lihuan Zeng, Hualin Fan, Ling Xue, Simin Liu, Jiyan Chen, Ning Tan, Pengcheng He, Chongyang Duan

**Affiliations:** ^1^Department of Cardiology, Guangdong Cardiovascular Institute, Guangdong Provincial People’s Hospital, Guangdong Academy of Medical Sciences, Guangzhou, China; ^2^Guangdong Provincial Key Laboratory of Coronary Heart Disease Prevention, Guangdong Provincial People’s Hospital, Guangdong Academy of Medical Sciences, Guangzhou, China; ^3^Department of Cardiology, Shanghai Institute of Cardiovascular Diseases, Zhongshan Hospital, Fudan University, Shanghai, China; ^4^Department of Cardiology, The Second People’s Hospital of Nanhai District, Guangdong General Hospital’s Nanhai Hospital, Foshan, China; ^5^Department of Cardiology, Second Clinical Medical College of Jinan University, Shenzhen People’s Hospital, First Affiliated Hospital of South University of Science and Technology, Shenzhen, China; ^6^Clinical College of Fujian Provincial Hospital, Fujian Provincial Hospital, Fujian Provincial Key Laboratory of Cardiovascular Disease, Fujian Provincial Center for Geriatrics, Fujian Medical University, Fujian Cardiovascular Institute, Fuzhou, China; ^7^Department of Cardiology, People’s Hospital of Baoan Shenzhen, Shenzhen, China; ^8^Center for Global Cardiometabolic Health, Department of Epidemiology, Medicine, and Surgery, Brown University, Providence, RI, United States; ^9^The Second School of Clinical Medicine, Southern Medical University, Guangzhou, China; ^10^Department of Biostatistics, School of Public Health, Southern Medical University, Guangzhou, China

**Keywords:** risk score, infection, ST-elevation myocardial infarction, percutaneous coronary intervention, observational study

## Abstract

**Background:**

Infection during hospitalization is a serious complication among patients who suffered from acute myocardial infarction (AMI) undergoing percutaneous coronary intervention (PCI); however, there are no suitable and accurate means to assess risk. This study aimed to develop and validate a simple scoring system to predict post-AMI infection in such patients.

**Methods:**

All patients with ST-segment elevation myocardial infarction (STEMI) undergoing PCI consecutively enrolled from January 2010 to May 2016 were served as derivation cohort, and those from June 2016 to May 2018 as validation cohort, respectively. The primary endpoint was post-AMI infection during hospitalization, and all-cause death and major adverse cardiovascular events (MACE) were considered as secondary endpoints. The simplified risk model was established using logistic regression. The area under the receiver operating curve and calibration of predicted and observed infection risk were calculated.

**Results:**

A 24-point risk score was developed, with infection risk ranging from 0.7 to 99.6% for patients with the lowest and highest score. Seven variables including age, Killip classification, insulin use, white blood cell count, serum albumin, diuretic use, and transfemoral approach were included. This model achieved the same high discrimination in the development and validation cohort (C-statistic:0.851) and revealed adequate calibration in both datasets. The incidences of post-AMI infection increased steadily across risk score groups in both development (1.3, 5.1, 26.3, and 69.1%; *P* < 0.001) and validation (1.8, 5.9, 27.2, and 79.2%; *P* < 0.001) cohort. Moreover, the risk score demonstrated good performance for infection, in-hospital all-cause death, and MACE among these patients, as well as in patients with the non-ST-elevation acute coronary syndrome.

**Conclusion:**

This present risk score established a simple bedside tool to estimate the risk of developing infection and other in-hospital outcomes in patients with STEMI undergoing PCI. Clinicians can use this risk score to evaluate the infection risk and subsequently make evidence-based decisions.

## Introduction

Infection during hospitalization is a severe complication among patients with acute myocardial infarction (AMI) (1, 2), which increases mortality risk, prolongs hospital stay, and drives up costs (3, 4). Considering these growing concerns, infection prevention is one of the highest priorities for healthcare payers and providers. Since a substantial proportion of infection is considered preventable, it is urgent to accurately and efficiently identify the risk of infection in patients with AMI and to develop an intervention for infection prevention.

Several mortality risk scores have been proposed for patients with ST-segment elevation myocardial infarction (STEMI) to date (5, 6), however, few of them have been validated for the post-AMI infection prediction. Although some other risk scores were developed to predict infection after cardiac surgery (7, 8), no study has established a simplified scoring model yet to estimate an individual patient’s risk of infection in patients with STEMI after cardiac catheterization. Therefore, we aimed to develop and validate a simple-to-use risk model to identify patients with STEMI at risk of post-AMI infection.

## Materials and Methods

### Study Population

The patient population used to develop this risk score (development cohort) consisted of the eligible patients enrolled in Guangdong Provincial People’s Hospital between January 2010 and May 2016. All patients with STEMI underwent PCI *via* radial, femoral, or both approaches, while patients with both accesses were classified as those with femoral assess, and patients with implantation of intra-aortic balloon pump (IABP) were also included. According to the same criteria, the population used for the validation (validation cohort) included those enrolled between June 2016 and May 2018, with complete data compared to the development cohort. Additionally, the patients screened in accordance with the criteria in other research centers were included for external validation. To widen the extent of application, this risk model was also validated among patients with the non-ST-elevation acute coronary syndrome (NSTE-ACS) treated with PCI in our recent publication (9).

Other details of the patients, study design, treatment and procedure, and outcome definitions have been addressed in our previously published protocol (10). Clinical data including patient demographics, laboratory tests, procedural details, medical treatments, and clinical outcomes were collected from the medical records. Vital variables (such as clinical events) were double-checked, and inconsistent data were verified by a third researcher. All clinical adverse events were evaluated by an independent clinical events committee blinded to the research.

### Study Endpoints

The primary endpoint was the occurrence of a P-AMI infection during hospitalization, which was defined as the infection requiring antibiotics (reflecting the clinical influence of infection compatible with the necessity for additional treatment) (11). Besides, the infection was determined using ICD-10-CM codes. The types of infections included pulmonary infection, urinary infection, or other infections including abdominal sepsis, primary bacteremia, and unidentified primary infection site based on the clinical records. Other details were reported in the published protocol (10). The secondary endpoints were in-hospital all-cause death, and major adverse cardiovascular events (MACE) including stroke, all-cause death, target vessel revascularization, and recurrent myocardial infarction.

### Sample Size

As for sample size determination, the concept of events per variable (EPV) of 10 or greater was applied as previously reported (12). Considering that no more than 8 factors were included in the development model, at least 80 infections were required. Our development dataset comprised 296 infections, and the value of events per variable was 42.3 (296/7).

### Statistical Analysis

Categorical variables were expressed as number (percentage) and continuous variables were expressed as mean ± SD, as appropriate. We chose a backward stepwise logistic regression model to develop the risk score. All listed candidate predictors (Supplementary Table 1) were included to develop the risk score, while variables with ≥ 10% missing values were excluded. And for those with incomplete data, we assumed that patients with the missing data ≤ 5% occurred at random depending on the clinical variables and used multiple imputations. Besides, a bootstrap method was applied to pick out the best subset of risk factors.

The predictive accuracy was evaluated using both discriminations evaluated by the C-statistic, and calibration assessed by the Hosmer-Lemeshow χ^2^ statistic and calibration plot. We performed the external and subgroup validation (older, sex, anemia, diabetes, and chronic kidney disease in the development and validation cohorts) to further measure the risk score’s stability. Additionally, we compared the discrimination of the present model with other risk scores previously reported using the area under the receiver operating characteristic (13–15). To assess the utility of the risk score, we conducted the decision curve analysis. A higher net benefit across the whole range of clinically applicable and acceptable probability thresholds indicated better clinical values (16). Other detailed information of the statistical analysis has been previously published (10).

All analyses were performed with SAS (version 9.4, SAS Institute, 210 Cary, NC, United States) and all figures were plotted with R (version 3.6.2, R Foundation for Statistical Computing, Vienna, Austria).

## Results

### Characteristic of the Development Cohort

**FIGURE 1:**
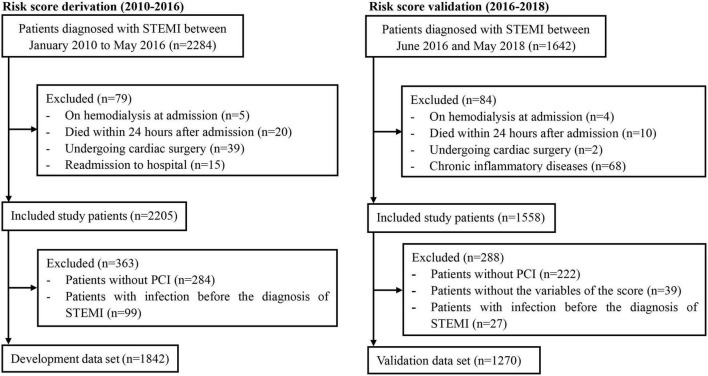
Flow diagram of the included population.

A total of 1,842 patients with STEMI who underwent PCI (mean age, 61.54 years; 17.5% female) were finally included in the development cohort (Figure 1). Among them, 217 patients (11.8%) experienced post-AMI infection during hospitalization. The in-hospital all-cause death was 17.5%, compared to 1.9% for patients without infections. Patients with post-AMI infection were more likely to be older, and have diabetes, chronic obstructive pulmonary disease (COPD), higher white blood cell (WBC) counts, and diuretics or insulin use. Moreover, these patients had lower levels of serum albumin, hemoglobin, estimated glomerular filtration rate, and left ventricular ejection fraction (Table 1).

**TABLE 1:** Baseline characteristics of included patients in the development cohort and validation cohort.

Variables	Development cohort		*P*-value	Validation cohort		*P*-value
	Infection (*n* = 217)	No infection (*n* = 1,625)		Infection (*n* = 142)	No infection (*n* = 1,128)	
Age, (years)	67.74 ± 11.94	60.71 ± 12.12	<0.001	67.96 ± 11.85	61.22 ± 11.91	<0.001
Age > 75 year, n (%)	135 (62.2%)	635 (39.1%)	<0.001	90 (63.4%)	453 (40.2%)	<0.001
Male, n (%)	177 (81.6%)	1,342 (82.6%)	0.711	110 (77.5%)	926 (82.1%)	0.180
Systolic blood pressure, (mmHg)	114.83 ± 23.90	122.90 ± 21.61	<0.001	114.58 ± 19.92	123.84 ± 20.68	<0.001
Diastolic blood pressure, (mmHg)	69.26 ± 13.27	74.11 ± 13.06	<0.001	71.29 ± 15.50	74.93 ± 13.06	0.008
Heart rate (bpm)	86.24 ± 21.23	78.85 ± 14.84	<0.001	86.75 ± 17.49	77.53 ± 13.66	<0.001
**Killip class, n (%)**						
I	82 (37.8%)	1263 (77.7%)	<0.001	58 (40.8%)	840 (74.5%)	<0.001
II	74 (34.1%)	283 (17.4%)		33 (23.2%)	240 (21.3%)	
III	29 (13.4%)	46 (2.8%)		31 (21.8%)	32 (2.8%)	
IV	32 (14.7%)	33 (2.0%)		20 (14.1%)	16 (1.4%)	
**Medical history, n (%)**						
Hypertension	124 (57.1%)	811 (49.9%)	0.045	82 (57.7%)	559 (49.6%)	0.066
Diabetes	70 (32.3%)	396 (24.4%)	0.012	47 (33.1%)	336 (29.8%)	0.418
Current smoker	80 (36.9%)	741 (45.6%)	0.015	40 (28.2%)	439 (39.0%)	0.012
Prior myocardial infraction	12 (5.5%)	76 (4.7%)	0.580	64 (45.1%)	690 (61.2%)	<0.001
Prior coronary artery bypass graft	1 (0.5%)	2 (0.1%)	0.246	0 (0.0%)	1 (0.1%)	0.723
COPD	10 (4.6%)	25 (1.5%)	0.002	2 (1.4%)	18 (1.6%)	0.866
Atrial fibrillation	13 (6.0%)	40 (2.5%)	0.003	12 (8.5%)	31 (2.7%)	<0.001
Prior stroke	28 (12.9%)	83 (5.1%)	<0.001	19 (13.4%)	63 (5.6%)	<0.001
**Laboratory characteristics**						
eGFR, (mL/min/1.73 m^2^)	63.42 ± 31.73	88.76 ± 30.48	<0.001	59.76 ± 28.04	82.01 ± 26.81	<0.001
Creatine kinase MB, (U/L) (IQR)	25.4 (12.3–64.3)	39.2 (14.1–84.4)	0.005	26.7 (10.5–124.63)	39.35 (13.25–276.03)	0.004
Serum creatinine, (mg/dL)	1.62 ± 1.40	1.06 ± 0.69	<0.001	1.70 ± 1.77	1.14 ± 0.82	<0.001
Serum albumin, (g/L)	31.29 ± 4.31	33.86 ± 3.94	<0.001	33.13 ± 3.90	36.59 ± 3.95	<0.001
Random blood glucose, (mmol/L) (IQR)	7.28 (6.17–9.28)	8.32 (6.98–11.25)	<0.001	7.15 (5.97–9.69)	8.48 (7.02–11.39)	<0.001
White blood cell count, (10^9^/L)	14.17 ± 4.49	11.55 ± 3.58	<0.001	14.13 ± 5.17	10.56 ± 3.58	<0.001
Hemoglobin, (g/L)	130.91 ± 21.08	134.95 ± 22.84	0.014	124.75 ± 22.62	133.31 ± 17.66	<0.001
Triglycerides, (mmol/L)	1.46 ± 1.00	1.59 ± 1.18	0.077	1.46 ± 0.72	1.73 ± 1.03	<0.001
Total cholesterol, (mmol/L)	4.58 ± 1.28	4.94 ± 1.20	<0.001	4.73 ± 1.61	4.83 ± 1.26	0.449
Total bilirubin, (mmol/L)	20.69 ± 11.99	17.70 ± 7.18	<0.001	18.55 ± 8.80	14.99 ± 7.11	<0.001
Low-density lipoprotein, (mmol/L)	2.85 ± 1.06	3.17 ± 1.04	<0.001	3.18 ± 1.21	3.24 ± 0.97	0.535
High-density lipoprotein, (mmol/L)	0.96 ± 0.28	0.97 ± 0.25	0.344	1.00 ± 0.27	1.00 ± 0.30	0.982
LVEF, (%)	47.01 ± 12.87	53.37 ± 10.47	<0.001	42.40 ± 11.18	52.58 ± 11.21	<0.001
**Medication during hospitalization, n (%)**						
Glycoprotein IIb/IIIa inhibitors	152 (70.0%)	1,115 (68.6%)	0.669	85 (59.9%)	516 (45.7%)	0.001
Statins	212 (97.7%)	1,606 (98.8%)	0.166	125 (88.0%)	1,101 (97.6%)	<0.001
Acetylsalicylic acid	214 (98.6%)	1,604 (98.7%)	0.912	139 (97.9%)	1,112 (98.6%)	0.521
Clopidogrel	213 (98.2%)	1,607 (98.9%)	0.349	127 (90.1%)	1,043 (92.5%)	0.299
Warfarin	3 (1.4%)	16 (1.0%)	0.586	3 (2.1%)	18 (1.6%)	0.649
ACEI	154 (71.0%)	1,339 (82.4%)	<0.001	81 (57.0%)	742 (65.8%)	0.040
Calcium channel blockers	34 (15.7%)	138 (8.5%)	<0.001	14 (9.9%)	108 (9.6%)	0.914
Angiotensin receptor blockers	44 (20.3%)	226 (13.9%)	0.013	26 (18.3%)	189 (16.8%)	0.642
β-blockers	163 (75.1%)	1,410 (86.8%)	<0.001	95 (66.9%)	925 (82.0%)	<0.001
Insulin therapy	64 (29.5%)	188 (11.6%)	<0.001	37 (26.1%)	154 (13.7%)	<0.001
Metformin	6 (2.8%)	71 (4.4%)	0.267	5 (3.6%)	76 (6.8%)	0.147
Proton pump inhibitor	163 (75.1%)	1,075 (66.2%)	0.008	108 (76.1%)	855 (75.8%)	0.946
Diuretics	122 (56.2%)	331 (20.4%)	<0.001	66 (46.5%)	212 (18.8%)	<0.001
**Procedural characteristics**						
Radial access, n (%)	135 (62.2%)	1,426 (87.8%)	<0.001	90 (63.4%)	995 (88.2%)	<0.001
Femoral access, n (%)	82 (37.8%)	199 (12.2%)		52 (36.6%)	133 (11.8%)	
Multi-lesion, n (%)	172 (79.3%)	1095 (67.4%)	<0.001	115 (81.0%)	864 (76.6%)	0.241
Contrast volume, (mL) (IQR)	100 (100–150)	100 (100–150)	0.490	100 (82.5–150)	100 (90–132.5)	0.125
Number of stents, (n) (IQR)	1 (1–2)	1 (1–2)	0.204	1 (1–2)	1 (1–2)	0.855
Total length of stent, (mm) (IQR)	30 (18–50)	30 (21–46)	0.649	36 (23.75–58.5)	33 (22–54)	0.333
In-hospital stay, (d) (IQR)	6 (5–8)	12 (8–19)	<0.001	5 (4–7)	11 (8–14.25)	<0.001

*Values are mean ± SD, n (%) or median (interquartile range). ACEI, angiotensin-converting enzyme inhibitors; COPD, chronic obstructive pulmonary disease; eGFR, estimated glomerular filtration rate; IQR, interquartile range; LVEF, left ventricular ejection fraction.*

### Variable Identification and Risk Score Development

**FIGURE 2:**
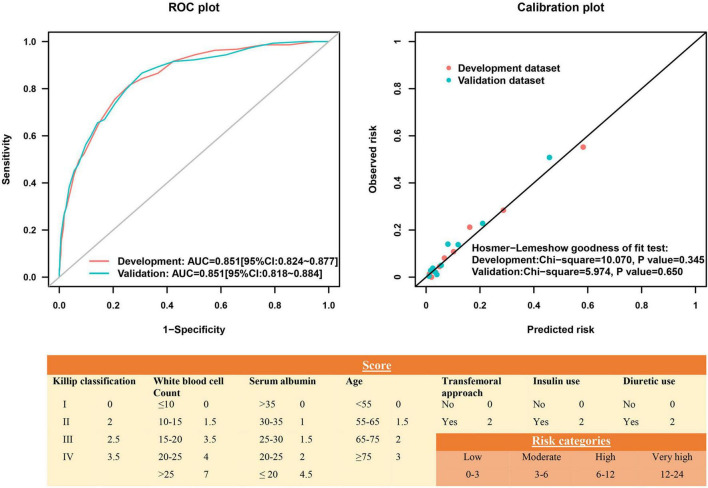
Receiver operating characteristic curve and calibration plot of the risk score for post-acute myocardial infarction (AMI) infection.

Among all 72 variables recorded according to the clinical data, 56 candidates were enrolled for the risk model development, while 16 variables were excluded for ≥ 10% missing values (Supplementary Table 1). After multivariate logistic regression analysis, the following seven variables were ultimately included: age, Killip classification, WBC count, serum albumin, insulin use, diuretic use, and transfemoral approach (Table 2 and Supplementary Figures 1, 2). Subsequently, the risk score, named post-AMI infection score (PAMIIS), was developed attributing corresponding points to each variable (Figure 2), and achieved predicted probabilities for infection development ranging from 0.7 to 99.6%, with a score range between 0 and 24 points (Supplementary Table 2 and Supplementary Figure 3).

**TABLE 2:** Univariate and multivariable logistic regression analysis of risk factors that were selected to develop the risk model for predicting post-AMI infection in the development cohort.

	Univariate analysis			Multivariable analysis			Multivariable analysis combined result based on 10 imputed data		
Variables	OR	95% CI	*P-*value	OR	95% CI	*P-*value	OR	95% CI	*P-*value
Age	1.05	1.04–1.07	<0.001	1.04	1.02–1.06	<0.001	1.04	1.02–1.05	<0.001
**Killip classification**									
I (reference)									
II	4.03	2.87–5.66	<0.001	2.05	1.39–3.01	<0.001	2.09	1.43–3.07	<0.001
III	9.71	5.80–16.26	<0.001	3.04	1.63–5.67	<0.001	2.77	1.51–5.08	0.001
IV	14.94	8.75–25.50	<0.001	5.38	2.83–10.26	<0.001	4.66	2.50–8.70	<0.001
White blood cell count	1.18	1.14–1.220	<0.001	1.16	1.12–1.21	<0.001	1.16	1.12–1.21	<0.001
Serum albumin	0.86	0.83–0.894	<0.001	0.92	0.88–0.95	<0.001	0.92	0.88–0.96	<0.001
Transfemoral approach	4.35	3.19–5.947	<0.001	2.58	1.78–3.76	<0.001	2.39	1.65–3.45	<0.001
Diuretics use	5.02	3.74–6.74	<0.001	2.35	1.65–3.34	<0.001	2.52	1.78–3.55	<0.001
Insulin use	3.20	2.30–4.44	<0.001	1.99	1.34–2.96	0.001	2.13	1.44–3.15	<0.001

*CI, confidence interval; OR, odds ratio.*

### Risk Score Internal Validation

**FIGURE 3:**
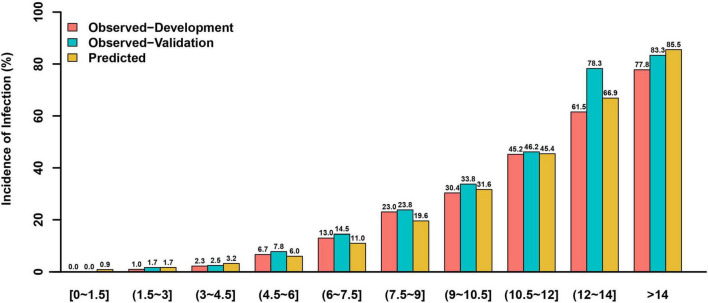
The observed and predicted incidences of post-AMI infection in the development and validation cohorts based on different risk scores.

This risk score showed good discriminative power (C-statistic:0.851, 95% confidence interval [CI]:0.824–0.877), with good calibration ability in line with the Hosmer-Lemeshow test (χ^2^ = 10.07; *P* = 0.345) (Figure 2), while the internal validation with bootstrapping also yielded a similar result (C-statistic:0.851, bias-corrected 95% CI:0.831–0.871). More notably, the calibration plots of predicted vs. observed incidences of post-AMI infection across risk scores confirmed an excellent calibration (Figure 3).

### Risk Score External Validation

For external validation, 1,270 patients with STEMI undergoing PCI were enrolled, and 142 patients (11.2%) experienced post-AMI infection, with a similar infection rate compared to the development cohort (11.2% vs. 11.8%; *P* = 0.607) (Table 1). Multiple differences in baseline characteristics were observed between the validation and development cohorts (Supplementary Table 3). The validation dataset exhibited semblable distribution of factors (excluding serum albumin, and WBC count) incorporated in the current risk model, and mean risk scores in comparison with the development dataset.

In the validation cohort, this model had similarly good discriminative power and calibration as the development cohort (C-statistic: 0.851, 95% CI:0.818–0.884; Hosmer-Lemeshow test χ^2^ = 5.974, *P* = 0.650), respectively (Figure 2). The observed incidences of post-AMI infection were highly consistent with the predicted ones (Figure 3).

Furthermore, after validation in the population from other two centers, in which 1,002 patients with STEMI undergoing PCI were included (Supplementary Figure 4) and 75 (7.5%) experienced post-AMI infection, this model remained a prominent predictor of infection (C-statistic:0.770, 95% CI:0.710–0.831; Hosmer-Lemeshow test χ^2^ = 15.412, *P* = 0.118) (Supplementary Figure 5).

### Risk Score Validation in Other Patients

To extend the utility, we further validated the risk score among patients with NSTE-ACS after PCI. A total of 4,745 patients (mean age, 63.70 years, 24.4% female, mean risk score 3.03 ± 2.15) were included (Supplementary Figure 6), and 118 (2.49%) experienced infection. The incidence of all-cause death and MACE were 0.30 and 0.80%, respectively. The comparison of baseline data was summarized between patients with or without infection in Supplementary Table 4. This risk model demonstrated analogously favorable discriminative ability in patients with NSTE-ACS (C-statistic:0.800, 95% CI:0.754–0.845), but the predicted risk was higher than the observed one in these relatively low-risk patients (Supplementary Figures 7, 8). After times 0.684 to the predicted risk, the observed incidences of post-AMI infection were highly consistent with the predicted ones with Hosmer-Lemeshow test χ^2^ value of 5.596 (*P* = 0.588, Supplementary Figure 7).

### Risk Score Predictive Values for Other In-Hospital Outcomes

Furthermore, this risk score exhibited good predictive values for in-hospital all-cause death and MACE in both cohorts. Similar results were observed among patients with NSTE-ACS (Supplementary Figure 9).

### Subgroup Analysis and Comparison With Other Risk Scores

As for IABP, there were 196 patients with implantation of IABP in the development cohort. To evaluate the potential influence of IABP on the risk score, we performed the subgroup of IABP analysis. And the result showed that the present risk score showed a similar predictive effect on infection between patients with (AUC:0.816, 95% CI:0.779–0.854) and without (AUC:0.789, 95% CI:0.724–0.854) use of IABP (Supplementary Table 5). Besides, subgroup analyses including age, gender, anemia, diabetes, and chronic kidney disease proved consistent predictive values for P-AMI infection in both datasets (Supplementary Table 5), with the best calibration in the aforementioned subgroups (calibration slope nearly 1) (Supplementary Figure 10).

Additionally, compared to previously established risk scores [age, estimated glomerular filtration rate, and ejection fraction (AGEF); age, creatinine, and ejection fraction (ACEF); and Canada Acute Coronary Syndrome (CACS) risk score], the present risk score, PAMIIS, achieved better discriminative power (Supplementary Figure 11).

### Clinical Use

The optimal cut-off identifying post-AMI infection was a score of 6.5 (sensitivity 81.5%; specificity 74.0%). As shown in Supplementary Figures 12, 13, increased risk of infection was obtained across risk score groups. Compared to those with a low-risk score, high-risk patients also presented elevated MACE and all-cause death in the development, validation, and NSTE-ACS cohorts (*P* < 0.001). Based on the types of infection, the incidences of pulmonary and urinary infection performed as the upward trend across groups in both datasets (Supplementary Figure 14). Decision curve analysis demonstrated that the present risk score displayed a better-standardized net benefit for infection than other risk scores, indicating better clinical usefulness (Supplementary Figure 15).

## Discussion

The present study is the first to develop a readily implementable, simple, and robust risk score including seven easily acquired parameters for post-AMI infection detection among patients with STEMI in clinical routine. Importantly, this risk score was validated in an independent validation cohort, as well as in various subgroups.

In the current study, the incidence of post-AMI infection was 11.8%, slightly higher than previously reported (10 and 3.9%) (4, 17). The elevated infection rate might be related to the prolonged median intensive care unit stay (4 days vs. 1 day) (18). However, the incidence was lower than a recent study including the octogenarian cohort undergoing primary PCI (28.9%) (19). In this regard, the different infection definitions and study populations might contribute to this discrepancy.

At present, several studies have reported predictive markers or established risk scores for infection in patients with cardiac surgery or critical illnesses. Fowler et al. identified and validated a risk model of 12 variables to detect cardiac surgery patients at high risk of major infections (8). Moreover, several predictive models have been validated to assess the infection risk in critically ill patients, such as the intensive care infection score (20). Yet, little research has been published on infection prediction models for patients with STEMI. Although our previous research has validated and compared the predictive significance of several clinical risk scores for post-AMI infection (13–15), these scores had relatively poor discriminatory powers and were not established specifically for infection. The present study is the first to develop and validate a risk model to predict infection in such patients and performed well across a broad range of patient subgroups.

Notably, diabetes mellitus (DM) generally causes an increase in susceptibility to infection among diabetics compared to those without DM (21). In the current study, we also documented that DM was an important risk factor of infection for patients with STEMI. However, DM with insulin therapy displayed better predictive value for post-AMI infection than DM *per se*. This observation is consistent with the previous results that patients with insulin therapy had a greater hazard of infection (22). Therefore, insulin use was incorporated into the final risk model instead of DM. In patients with type 2 DM, metformin is routinely used as the first-line oral drug; with disease progression, the vast majority of patients eventually have to use insulin to keep blood glucose levels within the desired range (23). Insulin therapy thus might represent the increased severity of DM to some extent, which would be more prone to develop infection (24). To better understand the potential mechanisms, further research should be performed to evaluate the relationship between insulin therapy and infection in detail.

Additionally, several risk factors (age and WBC count, etc.), significantly associated with infection by the multivariable analysis, were largely similar to risk factors previously identified. Serum albumin levels, an easily available biomarker, have been proven to be related to postoperative infections (25). Besides, findings from a systemic review demonstrated that the transradial PCI approach in STEMI patients significantly reduced major and access site bleeding, as well as the length of hospital stay, that was associated with the risk of infection (26). Importantly, hemorrhage is known to cause increased susceptibility to infection. Experimental studies also demonstrated that even a simple hemorrhage produced a significant depression of cellular immunity and the immunosuppression increased the susceptibility to sepsis (27). Essebag et al. also reported that clinically significant pocket hematomas were associated with a significantly increased infection risk in patients with cardiac implantable electronic device surgery (28). Thus, it is reasonable that the parameter transfemoral approach was finally included in the present risk score.

Importantly, it is widely recognized that the Killip classification and the diuretic use reflect the status of cardiac function. An increasing number of studies have demonstrated the complicated and vicious interactions between heart failure and infection (29, 30). The decline in cardiac function induces a rise in pulmonary pressure, subsequently leading to pulmonary edema and accumulation of pneumonia-related pathogens (Chlamydophila or streptococcus pneumonia), which in turn exhibit deleterious impacts on the respiratory infection (31). In addition, a recent study suggested that incident diuretic use markedly increased the emergency room visits or hospitalization rates related to pneumonia or COPD (32). However, due to the lack of relevant studies, the effects and underlying mechanisms of diuretics on infections remain unclear and need to be evaluated in further research.

The present risk score was based on simple, dichotomized factors, and thus can be rapidly and easily calculated in clinical practice. The identification of high-risk patients has important clinical implications, as several included factors are adaptable. Serum albumin levels and transfemoral PCI approaches can be modified, and more restrictive use of diuretic medications, as well as frequent blood glucose monitoring, are warranted to minimize the risk of infection. Additionally, this risk score might be helpful to select high-risk patients in future research to test interventions that reduce infections in such patients. Furthermore, although the present risk score was developed limitedly in patients with STEMI, the predictive value was also good in patients with NSTE-ACS. Thus, the utility of this risk score might be extended to all patients with ACS.

Several limitations should be mentioned. First, the score was exclusively developed in patients with STEMI and validated in patients with NSTE-ACS, but these findings should be used with caution when applied to other patients, such as critically ill patients. Second, infections are sometimes difficult to diagnose and might be overestimated occasionally. However, infections were defined as infections requiring antibiotic administration in this study to reduce misdiagnosis and overdiagnosis. Moreover, further study should be performed to evaluate the role of antibiotic type on outcomes. Third, the candidate variables for the risk-adjustment model were restricted to those available in our patient cohort, which would make the score less predictive accuracy than those with more included variables. Lastly, new biomarkers were not included in this risk score, while incorporating novel biomarkers would likely develop a more robust and productive scoring system. Noticeably, some inflammatory indicators, such as C-reactive protein and procalcitonin, were not included in this model since they were unconventional detection indexes, especially before the PCI procedure. Nonetheless, this risk-prediction instrument was established using commonly available clinical variables and would as such be more accessible clinically.

In summary, this study represents a simple-to-use risk score combining widely available clinical variables for infection estimation in patients with STEMI after PCI and can be applied in clinical routine. This scoring model might aid in risk stratification of infection and provoke future research into the best strategies to reduce or prevent infection development.

## Data Availability Statement

The raw data supporting the conclusions of this article will be made available by the authors, without undue reservation.

## Author Contributions

NT, PH, and YuL designed the study and revised the manuscript. LW, PC, YD, YaL, WC, ZX, LZ, and HF participated in the collection and assembly of data. CD and YuL did the statistical analysis. LW and YuL drafted the manuscript. All authors contributed to the critical revision of the article and approved the final manuscript.

## Conflict of Interest

The authors declare that the research was conducted in the absence of any commercial or financial relationships that could be construed as a potential conflict of interest.

## Publisher’s Note

All claims expressed in this article are solely those of the authors and do not necessarily represent those of their affiliated organizations, or those of the publisher, the editors and the reviewers. Any product that may be evaluated in this article, or claim that may be made by its manufacturer, is not guaranteed or endorsed by the publisher.
