# Supplementary material for: Risk Estimation for Infection in Patients With ST-Segment Elevation Myocardial Infarction Undergoing Percutaneous Coronary Intervention: Development and Validation of a Predictive Score
Source: Front Cardiovasc Med. 2022 Apr 15;9:845307. doi: 10.3389/fcvm.2022.845307 (PMC9051071; doi:10.3389/fcvm.2022.845307)
Supplement: Supplementary file 1 [file Data_Sheet_1.pdf]

Supplementary Tables .... **- 2 -**

Table S1. Candidate variables for the development of the risk score. .... - 2 -

Table S2. The scores and the predicted incidences of post-AMI infection.......... - 5 -

Table S3. Comparison of participant characteristics in the development and validation cohorts. .... - 7 -

Table S4. Baseline characteristics of patients with NSTE-ACS in the validation cohort.. - 10 -

Table S5. Risk score performance in subgroups. .... - 13 -

Supplementary Tables

Table S1. Candidate variables for the development of the risk score.

Variable	Number of missing values	Percentage (%)
**Included**		
**Demographics**		
Sex	0	0
Age	0	0
Systolic blood pressure	6	0.33%
Diastolic blood pressure	6	0.33%
Heart rate	0	0
Killip class	0	0
Hypertension	0	0
Diabetes	0	0
Smoke	0	0
Prior myocardial infraction	0	0
Coronary artery bypass graft	0	0
COPD	0	0
Prior Atrial fibrillation	0	0
Prior stroke	0	0
Prior PCI	0	0
Prior sudden cardiac arrest	0	0
Prior thrombolysis	0	0
Pericardial effusion	5	0.27%
**Laboratory characteristics**		
Estimated glomerular filtration rate	14	0.77%
Serum creatinine	14	0.77%
Serum albumin	76	4.30%
White blood cell count	0	0
Hemoglobin	0	0

Triglycerides	70	3.95%
Total cholesterol	70	3.95%
Total bilirubin	56	3.14%
Direct bilirubin	56	3.14%
Low-density lipoprotein	70	3.95%
High-density lipoprotein	70	3.95%
Left ventricular ejection fraction	85	4.84%
LVDd	110	6.35%
LVDs	149	8.80%
Urine PH	70	3.95%
CO_2 combining power	11	0.60%
Alanine aminotransferase	101	5.80%
**Medication during hospitalization**		
Glycoprotein IIb/IIIa inhibitors	0	0
Statins	0	0
Aspirin	0	0
Clopidogrel	0	0
Warfarin	0	0
Angiotensin receptor blockers	0	0
Calcium channel blockers	0	0
ACEI	0	0
β-blockers	0	0
Insulin therapy	0	0
Metformin	0	0
Proton pump inhibitor	0	0
Diuretics	0	0
Nitrates	0	0
**Procedural characteristics**		
PCI approach	0	0

Multi-lesion	0	0
Prior PCI lesion	0	0
Contrast volume	76	4.30%
Number of stents	0	0
Total length of stents	0	0
In-hospital day	0	0
**Excluded (missing ≥ 10%)**		
Time of admission	NA	NA
Time of discharge	NA	NA
T3	575	30%
T4	575	30%
Thyroid stimulating hormone	575	30%
Uric Acid	562	29%
Procalcitonin	1052	55%
Random blood glucose	365	19%
Preoperative NT-proBNP	1738	90%
Preoperative cystatin C	1861	97%
Preoperative Creatine kinase-MB	500	26%
Carotid artery stenosis	666	35%
Left carotid intima-media thickness	671	35%
Right carotid intima-media thickness	679	35%
Left carotid artery plaque	666	35%
Right carotid artery plaque	666	35%

Data are presented as the n (%) of subjects.

ACEI, angiotensin converting enzyme inhibitors; COPD, chronic obstructive pulmonary disease; LVDd, left ventricular end-diastolic diameter; LVDs, left ventricular end-systolic diameter; NT-proBNP, N-terminal-pro hormone B-type natriuretic peptide; PCI, percutaneous coronary intervention.

Table S2. The scores and the predicted incidences of post-AMI infection.

**Scores**	**Risks (%)**
0	0.7%
0.5	0.9%
1	1.1%
1.5	1.4%
2	1.7%
2.5	2.1%
3	2.6%
3.5	3.3%
4	4.0%
4.5	5.0%
5	6.1%
5.5	7.5%
6	9.1%
6.5	11.1%
7	13.4%
7.5	16.1%
8	19.3%
8.5	22.9%
9	27.0%
9.5	31.5%
10	36.4%
10.5	41.5%
11	46.9%
11.5	52.3%
12	57.7%
12.5	62.9%
13	67.8%

13.5	72.4%
14	76.5%
14.5	80.2%
15	83.4%
15.5	86.2%
16	88.6%
16.5	90.6%
17	92.3%
17.5	93.7%
18	94.9%
18.5	95.9%
19	96.6%
19.5	97.3%
20	97.8%
20.5	98.2%
21	98.6%
21.5	98.8%
22	99.1%
22.5	99.2%
23	99.4%
23.5	99.5%
24	99.6%

Table S3. Comparison of participant characteristics in the development and validation cohorts.

Variables	Development Cohort (n=1842)	Validation Cohort (n=1270)	*P*-value
Age, (year)	61.54 ± 12.31	61.97 ± 12.09	0.336
Age > 75year, n (%)	770 (41.8%)	543 (42.8%)	0.597
Male, n (%)	1519 (82.5%)	1036 (81.6%)	0.524
Female, n (%)	323 (17.5%)	234 (18.4%)	
Systolic blood pressure, (mmHg)	121.95 ± 22.04	122.82 ± 20.80	0.268
Diastolic blood pressure, (mmHg)	73.54 ± 13.17	74.53 ± 13.40	0.042
Heart rate (bpm)	79.72 ± 15.90	78.56 ± 14.43	0.034
Killip class, n (%)			
I	1345 (73.0%)	898 (70.7%)	0.197
II	357 (19.4%)	273 (21.5%)	
III	75 (4.1%)	63 (5.0%)	
IV	65 (3.5%)	36 (2.8%)	
**Medical history, n (%)**			
Hypertension	935 (50.8%)	641 (50.5%)	0.875
Diabetes	466 (25.3%)	383 (30.2%)	0.003
Smoke	821 (44.6%)	479 (37.7%)	<0.001
Prior myocardial infraction	88 (4.8%)	754 (59.4%)	<0.001
Coronary artery bypass graft	3 (0.2%)	1 (0.1%)	0.520
COPD	35 (1.9%)	20 (1.6%)	0.498
Atrial fibrillation	53 (2.9%)	43 (3.4%)	0.420
Prior stroke	111 (6.0%)	82 (6.5%)	0.624
**Laboratory characteristics**			
eGFR, (mL/min/1.73 m^2)	85.77 ± 31.69	79.52 ± 27.83	<0.001
Serum creatinine, (mg/dL)	1.13 ± 0.83	1.20 ± 0.98	0.032
Serum albumin, (g/L)	33.55 ± 4.07	36.20 ± 4.09	<0.001

Variables	Development Cohort (n=1842)	Validation Cohort (n=1270)	*P*-value
White blood cell count, (10^9/L)	11.86 ± 3.79	10.96 ± 3.95	<0.001
Hemoglobin, (g/L)	134.48 ± 22.67	132.35 ± 18.47	0.004
Triglycerides, (mmol/L)	1.57 ± 1.16	1.70 ± 1.01	0.001
Total cholesterol, (mmol/L)	4.90 ± 1.21	4.82 ± 1.30	0.095
Total bilirubin, (mmol/L)	18.06 ± 7.97	15.39 ± 7.40	<0.001
LDL, (mmol/L)	3.13 ± 1.05	3.24 ± 1.00	0.005
HDL, (mmol/L)	0.97 ± 0.26	1.00 ± 0.30	0.005
LVEF, (%)	52.59 ± 10.99	51.43 ± 11.66	0.009
**Medication during hospitalization, n (%)**			
Glycoprotein IIb/IIIa inhibitors	1267 (68.8%)	601 (47.3%)	<0.001
Statins	1818 (98.7%)	1226 (96.5%)	<0.001
Aspirin	1818 (98.7%)	1251 (98.5%)	0.650
Clopidogrel	1820 (98.8%)	1170 (92.3%)	<0.001
Warfarin, n (%)	19 (1.0%)	21 (1.7%)	0.130
ACEI	1493 (81.1%)	823 (64.8%)	<0.001
Calcium channel blockers	172 (9.3%)	122 (9.6%)	0.801
Angiotensin receptor blockers	270 (14.7%)	215 (16.9%)	0.086
β-blockers	1573 (85.4%)	1020 (80.3%)	<0.001
Insulin therapy	252 (13.7%)	191 (15.0%)	0.286
Metformin	77 (4.2%)	81 (6.4%)	0.006
Proton pump inhibitor	1238 (67.2%)	963 (75.8%)	<0.001
Diuretics	453 (24.6%)	278 (21.9%)	0.080
**Procedural characteristics**			
Radial access, n (%)	1561 (84.7%)	1085 (85.4%)	0.597
Femoral assess, n (%)	281 (15.3%)	185 (14.6%)	
Multi-lesion, n (%)	1267 (68.8%)	979 (77.1%)	<0.001
Contrast volume, (mL)	100 (100-150)	100 (90-150)	<0.001

Variables	Development Cohort (n=1842)	Validation Cohort (n=1270)	*P*-value
Number of stents, (n)	1 (1-2)	1 (1-2)	0.002
Total length of stent, (mm)	30 (21-46)	33 (23-54)	<0.001

Values are mean ± SD, n (%) or median (interquartile range).

ACEI, angiotensin converting enzyme inhibitors; COPD, chronic obstructive pulmonary disease; eGFR, estimated glomerular filtration rate; HDL, high-density lipoprotein; LDL, low-density lipoprotein; LVEF, left ventricular ejection fraction.

Table S4. Baseline characteristics of patients with NSTE-ACS in the validation cohort.

	Validation dataset		
**Variables**	**Infection (n=118)**	**No infection (n=4627)**	***P*-value**
Age, (year)	70.23 ± 8.71	63.53 ± 10.33	<0.001
Age > 75year, n (%)	86 (72.9%)	2180 (47.1%)	<0.001
Male, n (%)	89 (75.4%)	3499 (75.6%)	0.961
Female, n (%)	29 (24.6%)	1128 (24.4%)	
Systolic blood pressure, (mmHg)	135.37 ± 22.04	133.35 ± 19.02	0.329
Diastolic blood pressure, (mmHg)	75.82 ± 10.78	77.06 ± 11.27	0.241
Heart rate (bpm)	76.78 ± 13.91	73.78 ± 10.87	0.022
Killip class, n (%)			
I	92 (78.0%)	4190 (90.6%)	<0.001
II	19 (16.1%)	387 (8.4%)	
III	6 (5.1%)	43 (0.9%)	
IV	1 (0.8%)	6 (0.1%)	
**Medical history, n (%)**			
Hypertension	87 (73.7%)	3000 (64.8%)	0.045
Diabetes	54 (45.8%)	1467 (31.7%)	0.005
Smoke	35 (29.7%)	1213 (26.2%)	0.401
Prior myocardial infraction	30 (25.4%)	741 (16.0%)	0.006
Coronary artery bypass graft	2 (1.7%)	61 (1.3%)	0.724
Atrial fibrillation	4 (3.4%)	119 (2.6%)	0.581
Prior stroke	12 (10.2%)	279 (6.0%)	0.064
**Laboratory characteristics**			
eGFR, (mL/min/1.73 m^2)	63.79 ± 26.42	81.85 ± 24.99	<0.001
Serum creatinine, (mg/dL)	1.39 ± 0.93	1.05 ± 0.70	<0.001
Serum albumin, (g/L)	32.75 ± 4.58	36.12 ± 3.80	<0.001
White blood cell count, (10^9/L)	9.33 ± 3.43	7.58 ± 2.08	<0.001

Variables	Validation dataset		*P*-value
	Infection (n=118)	No infection (n=4627)	
Hemoglobin, (g/L)	121.22 ± 20.34	133.29 ± 16.68	<0.001
Triglycerides, (mmol/L)	1.57 ± 1.57	1.70 ± 1.33	0.398
Total cholesterol, (mmol/L)	4.41 ± 1.10	4.50 ± 1.22	0.443
Low-density lipoprotein, (mmol/L)	2.71 ± 0.89	2.73 ± 1.00	0.822
High-density lipoprotein, (mmol/L)	0.96 ± 0.25	0.97 ± 0.26	0.598
LVEF, (%)	56.88 ± 12.86	61.84 ± 10.76	<0.001
**Medication during hospitalization, n (%)**			
Glycoprotein IIb/IIIa inhibitors	26 (22.0%)	473 (10.2%)	<0.001
Statins	117 (99.2%)	4532 (97.9%)	0.358
Aspirin	115 (97.5%)	4508 (97.4%)	0.984
Clopidogrel	118 (100.0%)	4591 (99.2%)	0.336
Warfarin	1 (0.8%)	17 (0.4%)	0.402
ACEI/ARB	93 (78.8%)	3634 (78.5%)	0.943
Calcium channel blockers	41 (34.7%)	978 (21.1%)	<0.001
β-blockers	102 (86.4%)	3915 (84.6%)	0.586
Insulin therapy	34 (28.8%)	352 (7.6%)	<0.001
Metformin	3 (5.6%)	308 (20.9%)	0.006
Proton pump inhibitor	88 (74.6%)	2068 (44.7%)	<0.001
Diuretics	47 (39.8%)	537 (11.6%)	<0.001
**Procedural characteristics**			
Radial access, n (%)	84 (71.2%)	4245 (91.7%)	<0.001
Femoral assess, n (%)	34 (28.8%)	382 (8.3%)	NA
Multi-lesion, n (%)	101 (85.6%)	3448 (74.5%)	0.006
Contrast volume, (mL)	100 (0-150)	100 (100-150)	0.026
Number of stents, (n)	2 (1-3)	2 (1-3)	0.149
Total length of stent, (mm)	46.5 (30-66.25)	42 (24-66)	0.177

Values are mean ± SD, n (%) or median (interquartile range).

ACEI, angiotensin converting enzyme inhibitors; ARB, angiotensin receptor blockers; eGFR, estimated glomerular filtration rate; LVEF, left ventricular ejection fraction.

Table S5. Risk score performance in subgroups.

Subgroup	Development AUC	95%CI Lower	95%CI Upper	Validation AUC	95%CI Lower	95%CI Upper
**IABP**						
Yes	0.816	0.779	0.854	0.827	0.781	0.874
No	0.789	0.724	0.854	0.650	0.545	0.754
**Age**						
Age < 65years	0.851	0.808	0.894	0.867	0.814	0.919
Age ≥ 65years	0.824	0.786	0.863	0.806	0.757	0.855
**Gender**						
Male	0.860	0.831	0.888	0.852	0.816	0.888
Female	0.823	0.757	0.889	0.852	0.774	0.929
**Diabetes**						
Yes	0.850	0.817	0.883	0.860	0.819	0.901
No	0.851	0.809	0.894	0.836	0.778	0.894
**Anemia**						
Yes	0.839	0.803	0.876	0.842	0.797	0.888
No	0.857	0.819	0.895	0.849	0.800	0.898
**Chronic kidney disease**						
Yes	0.832	0.794	0.870	0.818	0.767	0.868
No	0.792	0.742	0.843	0.828	0.773	0.882

AUC, area under the curve; CI, confidence interval, IABP, intra-aortic balloon pump.
