# Supplementary material for: Risk Estimation for Infection in Patients With ST-Segment Elevation Myocardial Infarction Undergoing Percutaneous Coronary Intervention: Development and Validation of a Predictive Score
Source: Front Cardiovasc Med. 2022 Apr 15;9:845307. doi: 10.3389/fcvm.2022.845307 (PMC9051071; doi:10.3389/fcvm.2022.845307)
Supplement: Supplementary file 2 [file Data_Sheet_2.pdf]

Supplementary Figures

Figure S1. Importance of the included variables in the predictive model for post-AMI infection. .... - 2 -
Figure S2. Model performance (AIC, -2 Log L and AUC) comparison between different cutoff selections.. - 4 -
Figure S3. The nomogram to estimate the risk of post-AMI infection. .... - 5 -
Figure S4. Study flow of the patients with STEMI in other centers. .... - 6 -
Figure S5. Receiver operating characteristic curve and calibration plots for the risk score for infection in patients with STEMI in other centers. .... - 7 -
Figure S6. Study flow of the patients with NSTE-ACS.. - 8 -
Figure S7. Receiver operating characteristic curve and calibration plots for the risk score for infection in patients with NSTE-ACS.. - 9 -
Figure S8. The observed and predicted incidence of post-AMI infection in patients with NSTE-ACS based on different risk scores. .... - 10 -
Figure S9. Receiver operating characteristic curves for the risk scores for in-hospital all-cause death and MACE in patients with STEMI and NST-ACS. .... - 11 -
Figure S10. Calibration plot for post-AMI infection in the subgroups. .... - 12 -
Figure S11. Comparison of the current risk score with previously established risk scores. .. - 13 -
Figure S12. Incidences of infection, in-hospital all-cause death and MACE in the development and validation cohorts based on different risk score groups. .... - 14 -
Figure S13. Incidences of infection, in-hospital all-cause death, and MACE in patients with NSTE-ACS based on different risk score groups.. - 15 -
Figure S14. The incidence of different types of infection in the development and validation data based on the different risk score groups.. - 16 -
Figure S15. Decision curves of the risk scores for post-AMI infection. .... - 17 -

Supplementary Figures

Figure S1. Importance of the included variables in the predictive model for post-AMI infection.

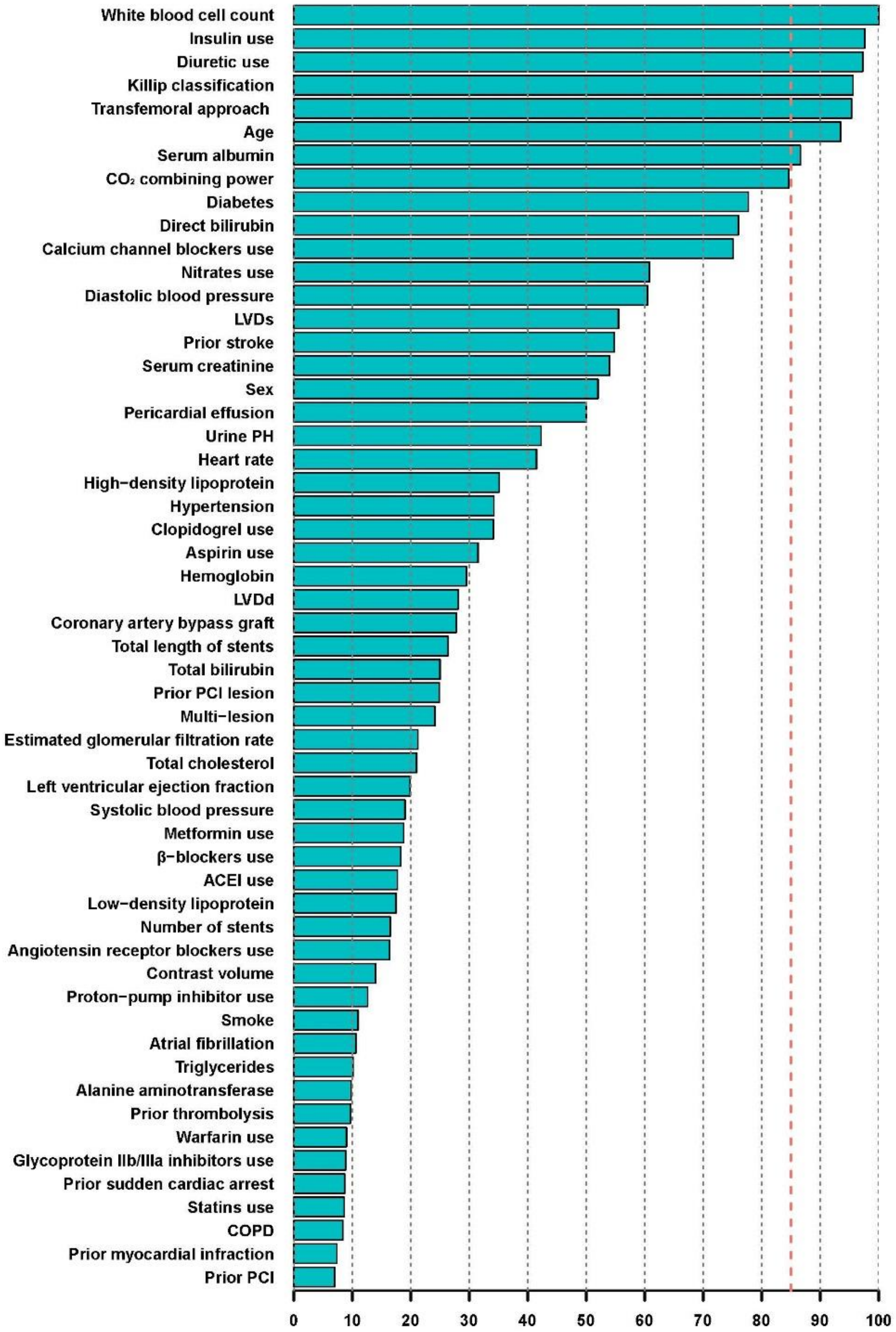

Variables that were selected in at least 85% of 1000 bootstrap repeats were included in the final multivariate models in this study. The mean proportion of variables being selected in 10 imputed datasets was displayed as in the above Figure S1. It should be pointed out that there is no standard procedure for the selection of cutoff. The cutoffs that have been used by researchers distributed from 60% to 90% (1-3). In our study, after we initially explored different cutoffs, setting 70%, 75%, 80%, 85%, 90%, and after tradeoff between model performance (see following Figure S2) and score ease-to-use, the 85% cutoff emerged as the most appropriate one.

Figure S2. Model performance (AIC, -2 Log L and AUC) comparison between different cutoff selections.

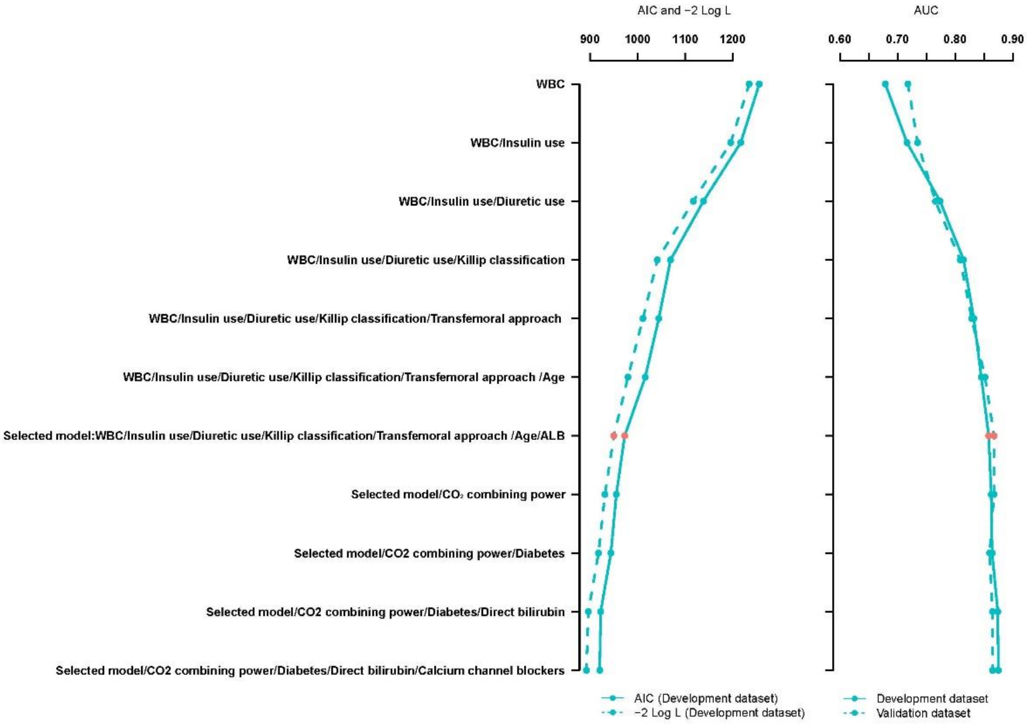

AIC, Akaike Information Criterion; ALB, albumin; AUC, area under the curve; WBC, white blood cell count.

Figure S3. The nomogram to estimate the risk of post-AMI infection.

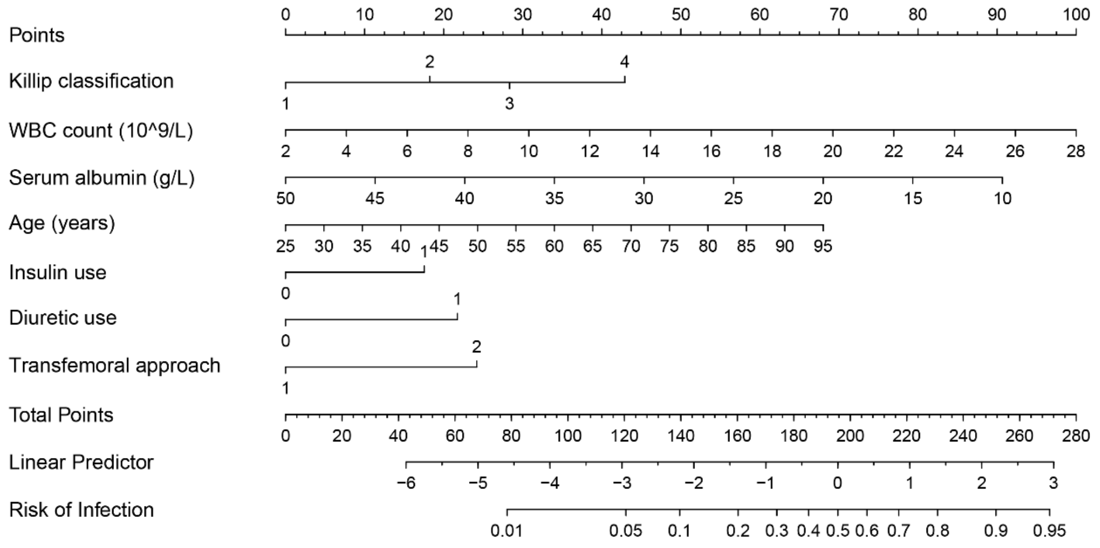

WBC, white blood cell count.

Figure S4. Study flow of the patients with STEMI in other centers.

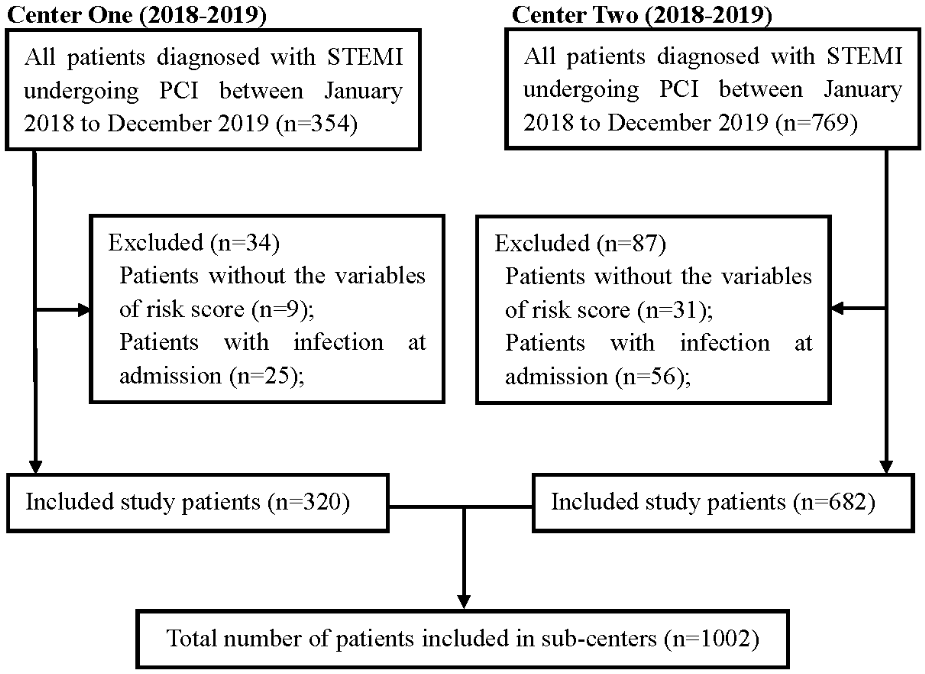

For external validation of the risk score, a total of 1002 STEMI patients undergoing PCI from two other centers between January 2018 and December 2019 were enrolled after exclusion of patients without the variables of the risk score and patients with infection at admission. PCI, percutaneous coronary intervention; STEMI, ST-segment elevation myocardial infarction.

Figure S5. Receiver operating characteristic curve and calibration plots for the risk score for infection in patients with STEMI in other centers.

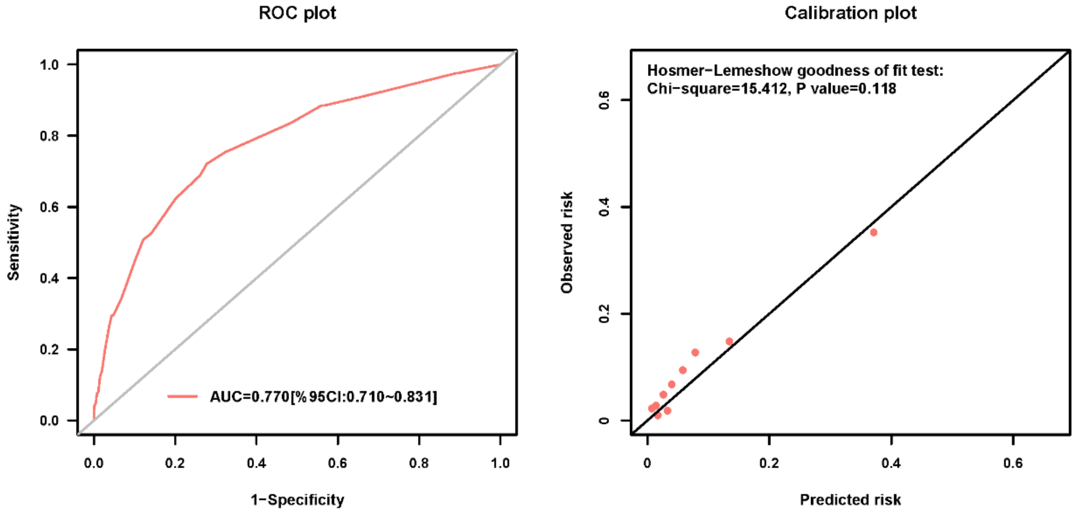

The risk score had good discriminative power, with a C-statistic of 0.770 and was well-calibrated according to the Hosmer-Lemeshow test (χ^2 = 15.412) for infection among STEMI patients in other centers. STEMI, ST-segment elevation myocardial infarction; AUC, area under the curve; CI, confidence interval; ROC, receiver operating characteristic.

Figure S6. Study flow of the patients with NSTE-ACS.

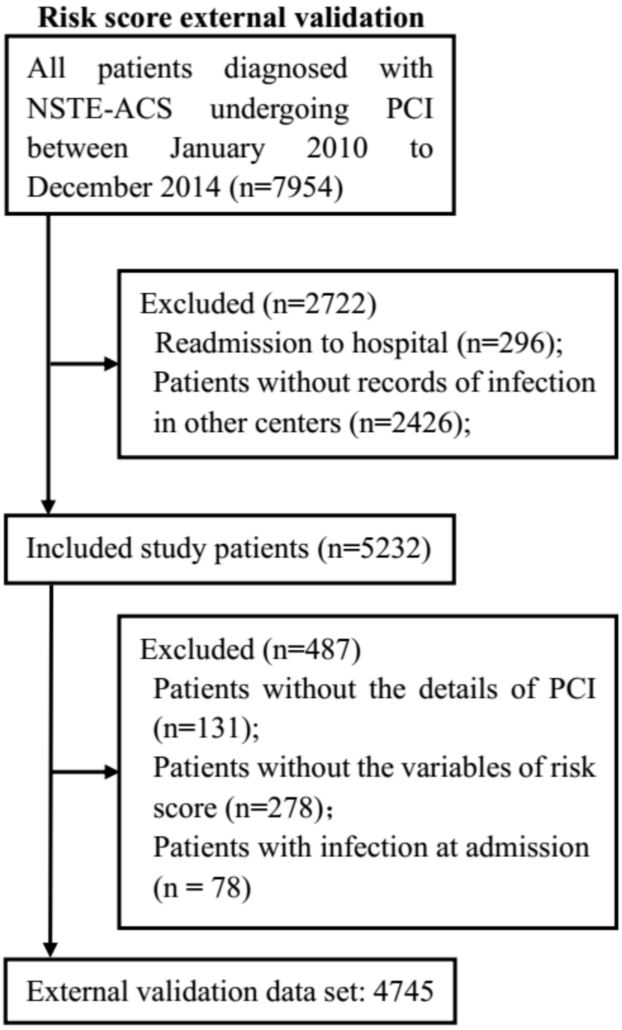

For external validation of the risk score, a total of 4745 NSTE-ACS patients undergoing PCI between January 2010 and December 2014 were enrolled according to the exclusion criteria. NSTE-ACS, non-ST-elevation acute coronary syndrome; PCI, percutaneous coronary intervention.

Figure S7. Receiver operating characteristic curve and calibration plots for the risk score for infection in patients with NSTE-ACS.

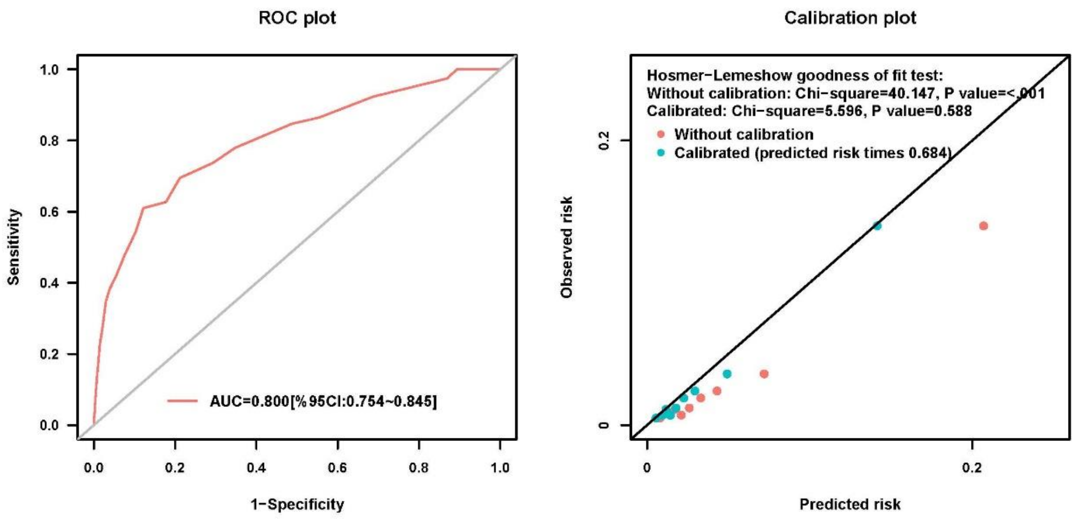

The risk score had a good discriminative power (C-statistic = 0.800) and was well-calibrated according to the Hosmer-Lemeshow test for infection among NSTE-ACS patients. NSTE-ACS, non-ST-elevation acute coronary syndrome; AUC, area under the curve; CI, confidence interval; ROC, receiver operating characteristic.

Figure S8. The observed and predicted incidence of post-AMI infection in patients with NSTE-ACS based on different risk scores.

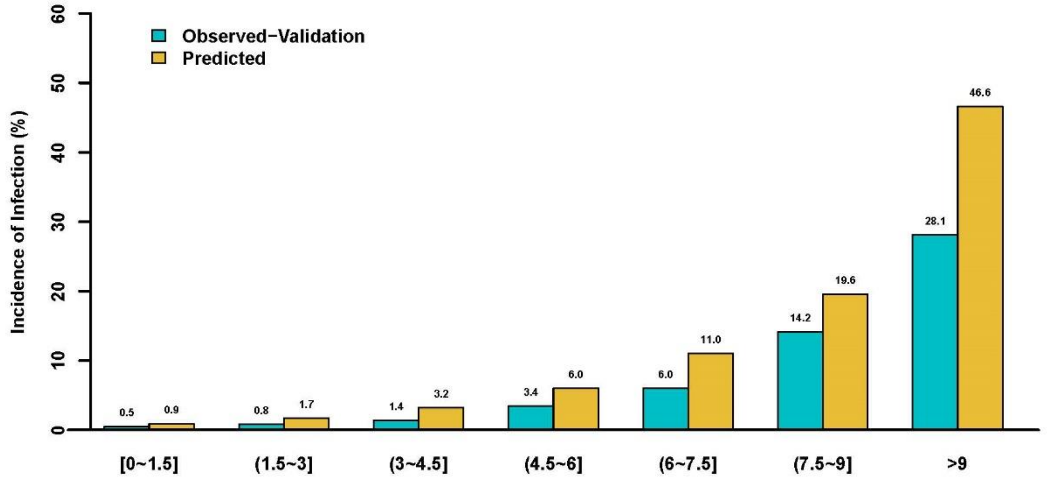

Increasing risk of infection with increasing risk score is evident in the validation data sets. And the observed incidence of infection was nicely consistent with the predicted ones using the risk score in NSTE-ACS patients. NSTE-ACS, non-ST-elevation acute coronary syndrome.

Figure S9. Receiver operating characteristic curves for the risk scores for in-hospital all-cause death and MACE in patients with STEMI and NST-ACS.

(A)

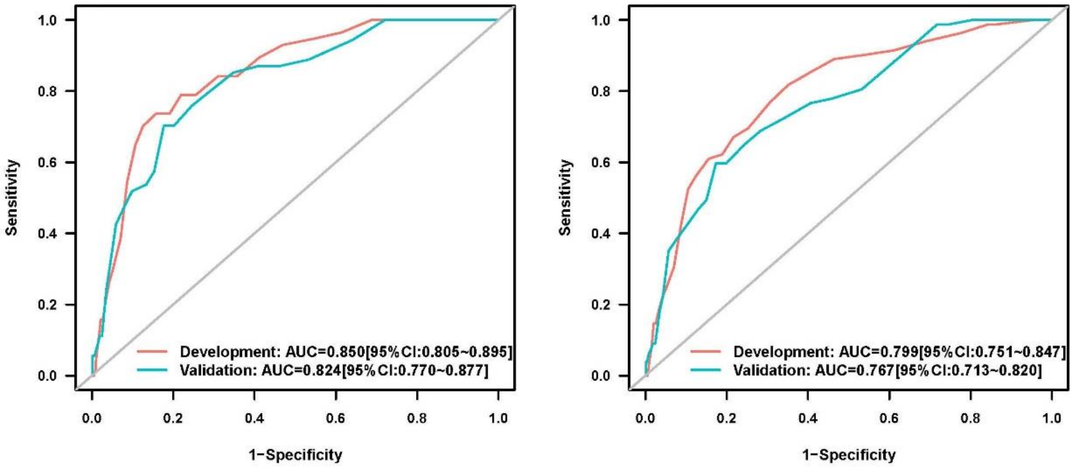

(B)

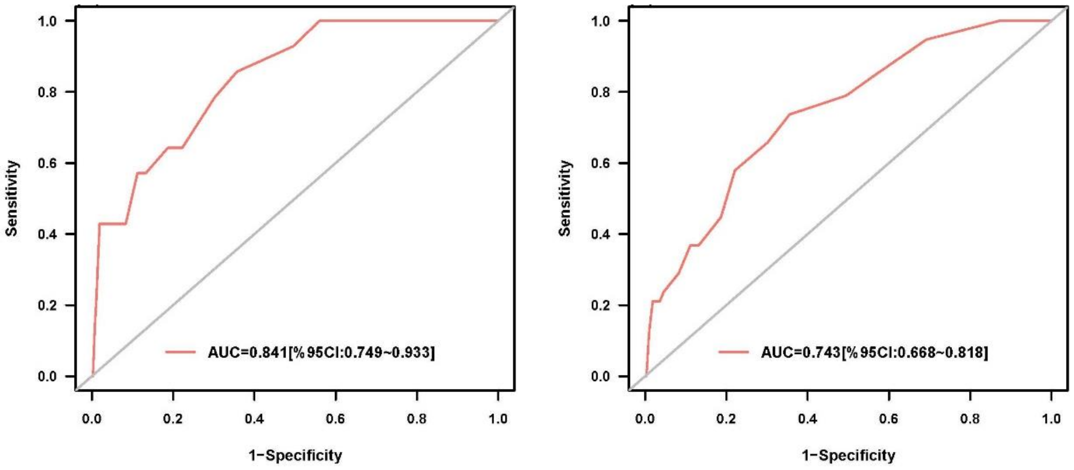

(**A**) The risk score achieved a good discriminative power for in-hospital all-cause death (left) and major adverse cardiovascular events (right) in both development and validation data sets among STEMI patients. (**B**) The risk score achieved a good discriminative power for in-hospital all-cause death (C-statistic = 0.841, left) and major adverse cardiovascular events (C-statistic = 0.743, right) among NSTE-ACS patients. AUC, area under the curve; CI, confidence interval.

Figure S10. Calibration plot for post-AMI infection in the subgroups.

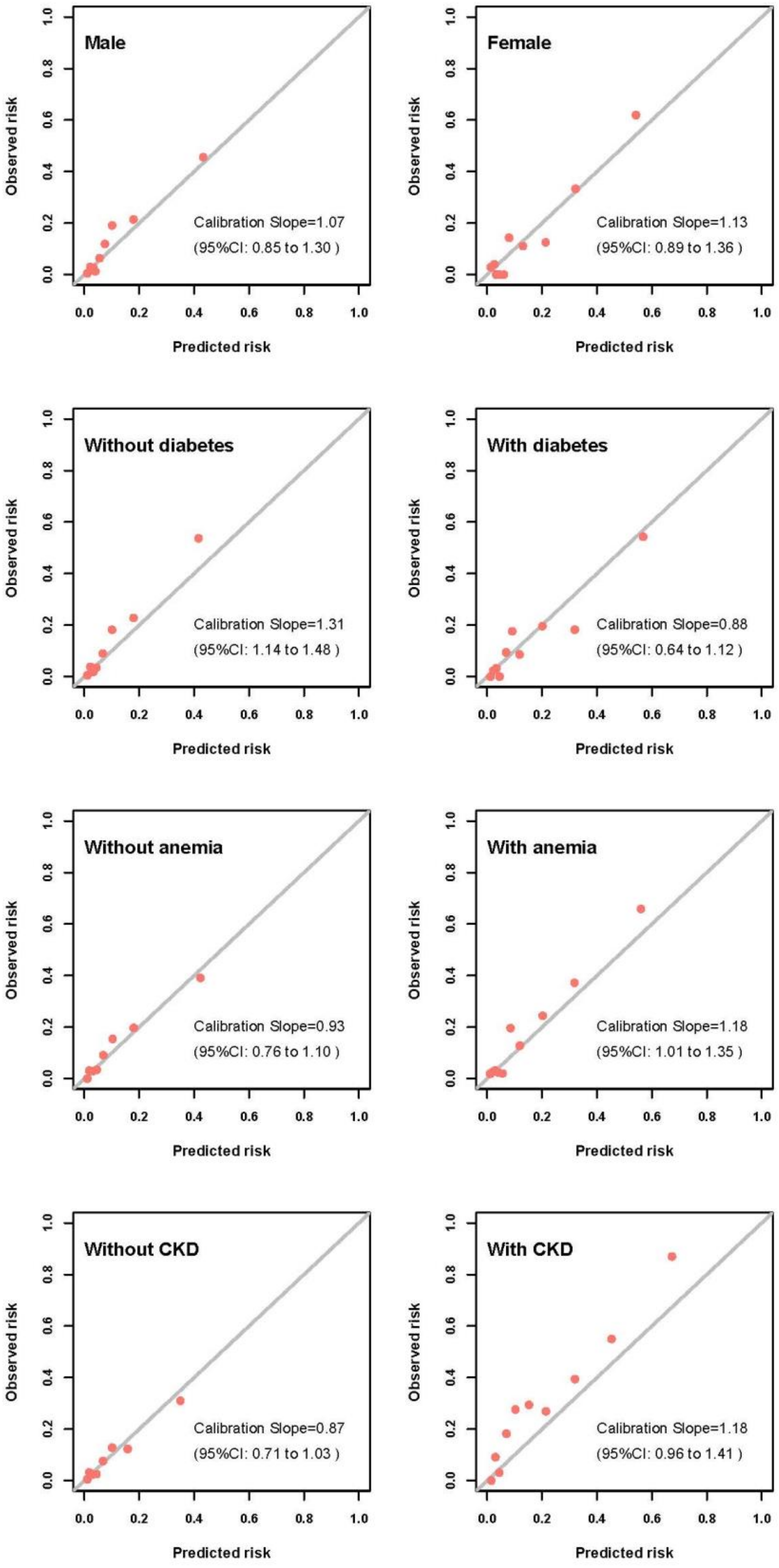

The risk score showed the best calibration for infection in subgroups (calibration slope nearly 1) .CI, confidence interval; CKD, chronic kidney disease.

Figure S11. Comparison of the current risk score with previously established risk scores.

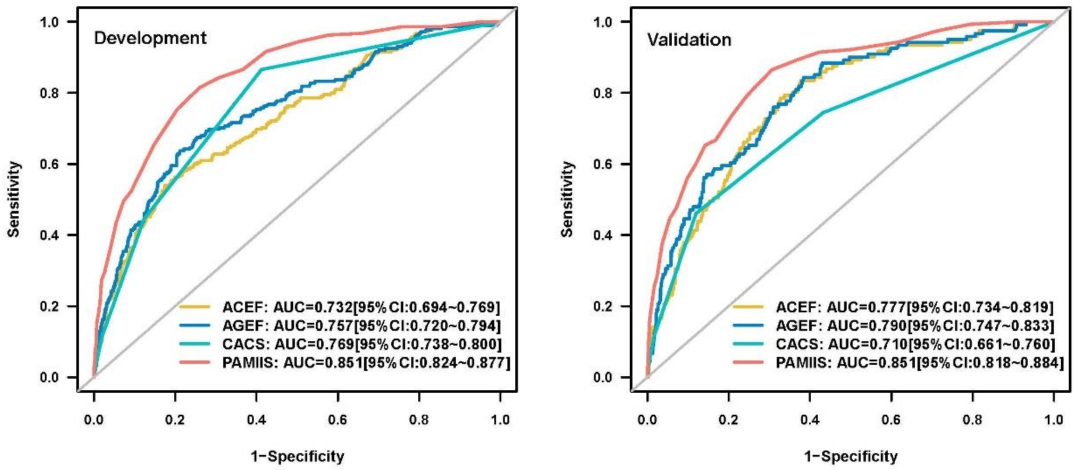

The PAMIIS score showed better discriminative power for infection in the development and validation cohorts compared with previously established risk scores. ACEF, Age, Creatinine, and Ejection Fraction; AGEF, Age, Glomerular Filtration Rate, and Ejection Fraction; AUC, area under the curve; CACS, Canada Acute Coronary Syndrome; CI, confidence interval; PAMIIS, Post-Acute Myocardial Infarction Infection Score.

Figure S12. Incidences of infection, in-hospital all-cause death and MACE in the development and validation cohorts based on different risk score groups.

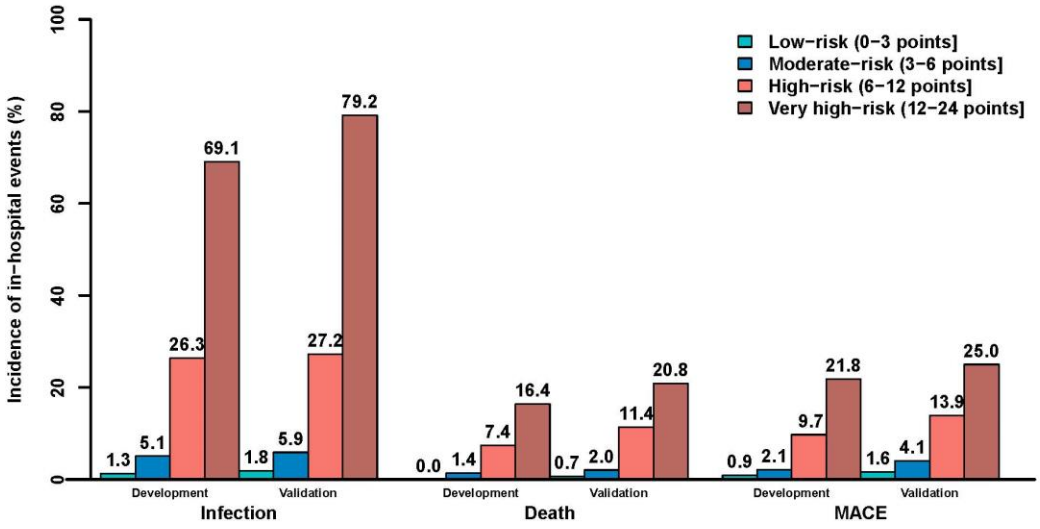

For definition of low (0-3], moderate (3-6], high (6-12] and very high (12-24] risk groups: The PAMIIS score derived from the development dataset predicted infection in the validation set, as well. And death and MACE can be predicted by the risk score value similarly in the development and validation datasets in STEMI patients. MACE, major adverse cardiovascular events.

Figure S13. Incidences of infection, in-hospital all-cause death, and MACE in patients with NSTE-ACS based on different risk score groups.

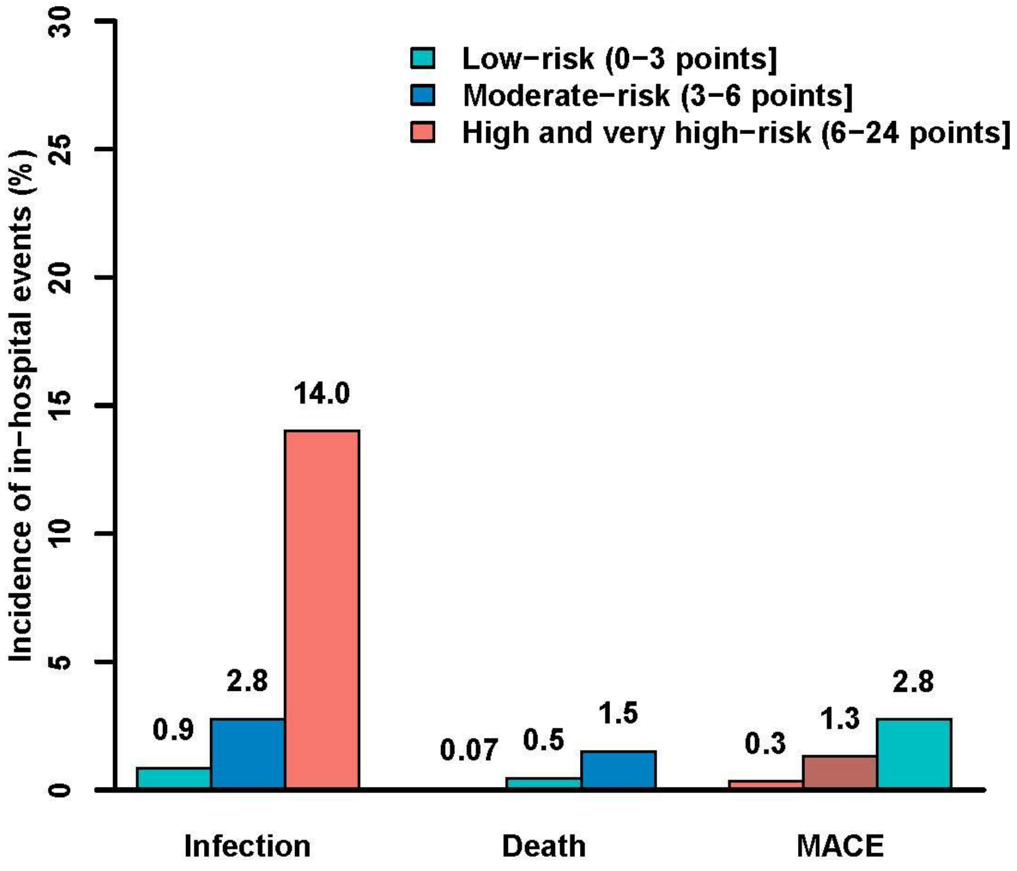

For definition of low (0-3], moderate (3-6], high and very high (6-24] risk groups: The PAMIIS score could predict infection, death and MACE in NSTE-ACS patients. MACE, major adverse cardiovascular events; NSTE-ACS, non-ST-elevation acute coronary syndrome.

Figure S14. The incidence of different types of infection in the development and validation data based on the different risk score groups.

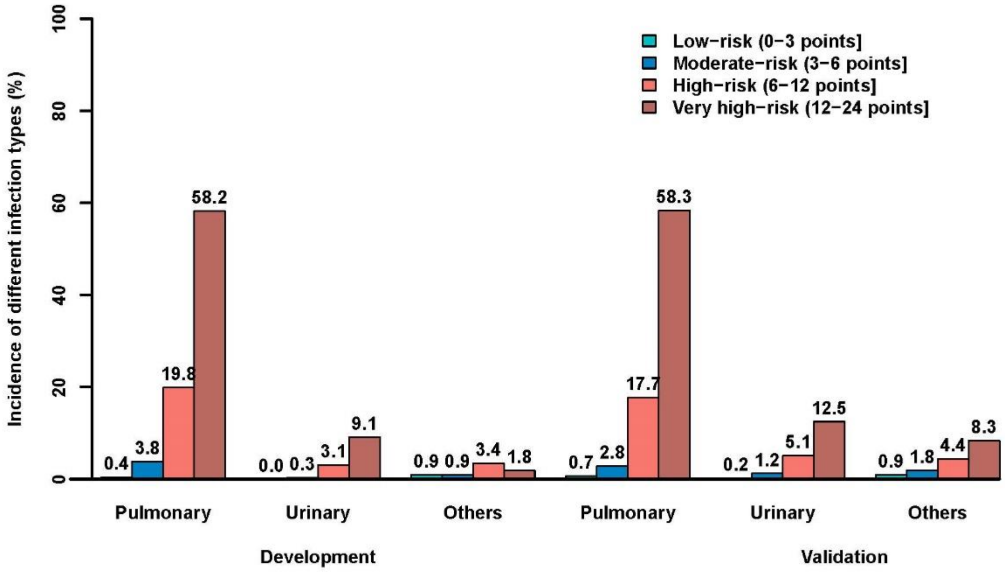

For definition of low (0-3], moderate (3-6], high (6-12] and very high (12-24] risk groups: The PAMIIS score derived from the development dataset could predict pulmonary, urinary and other infection in the development and validation sets in STEMI patients. MACE, major adverse cardiovascular events.

Figure S15. Decision curves of the risk scores for post-AMI infection.

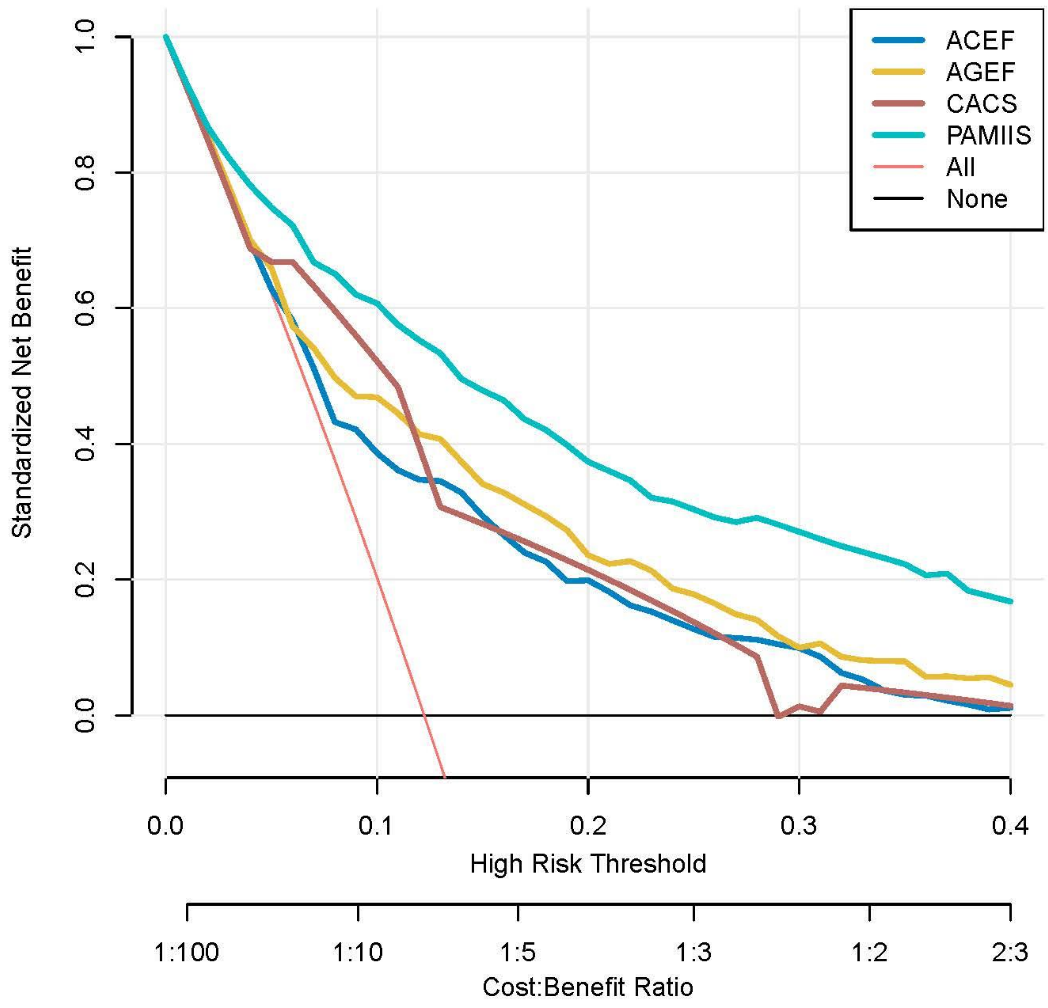

Decision curve analysis demonstrated that the PAMIIS score had a better standardized net benefit for infection than other risk scores, indicating better clinical usefulness. ACEF, Age, Creatinine, and Ejection Fraction; AGEF, Age, Glomerular Filtration Rate, and Ejection Fraction; CACS, Canada Acute Coronary Syndrome; PAMIIS, Post-Acute Myocardial Infarction Infection Score.

References

1. Austin PC, Tu JV. Bootstrap Methods for Developing Predictive Models. The American Statistician. 2004; 58:131-137.

2. Mehran R, Aymong ED, Nikolsky E, Lasic Z, Iakovou I, Fahy M, et al. A simple risk score for prediction of contrast-induced nephropathy after percutaneous coronary intervention. J Am Coll Cardiol. 2004. 44:1393-1399.

3. Goliasch G, Kleber ME, Richter B, Plischke M, Hoke M, Haschemi A, et al. Routinely available biomarkers improve prediction of long-term mortality in stable coronary artery disease: the Vienna and Ludwigshafen Coronary Artery Disease (VILCAD) risk score. Eur Heart J. 2012; 33: 2282-2289.
